# Lactic Acid Bacteria-Derived Postbiotics in Dairy Foods: Definitions, Functions and Regulatory Challenges

**DOI:** 10.3390/microorganisms14071520

**Published:** 2026-07-12

**Authors:** Evelina Mockute, Jurgita Aksomaitiene, Aleksandr Novoslavskij, Kristina Kondrotiene

**Affiliations:** Department of Food Safety and Quality, Faculty of Veterinary Medicine, Lithuanian University of Health Sciences, Tilzes Str. 18, LT-47181 Kaunas, Lithuania; evelina.mockute@lsmu.lt (E.M.); jurgita.aksomaitiene@lsmu.lt (J.A.); aleksandr.novoslavskij@lsmu.lt (A.N.)

**Keywords:** postbiotics, fermented milk products, LAB

## Abstract

Lactic acid bacteria (LAB) are essential in the production of fermented dairy products, contributing to desirable sensory attributes, safety, and shelf life. Traditionally, the health-promoting properties of fermented dairy products have been associated with live probiotic LAB; however, ensuring their viability during processing, storage and gastrointestinal passage remains challenging. This has shifted scientific interest toward postbiotics—preparations consisting of inactivated microbial cells, cell components and fermentation-derived metabolites capable of resulting in health benefits without the survival of microorganisms. LAB-derived postbiotics include bioactive peptides, exopolysaccharides and cell wall fragments, which may support gut barrier function, exert immunomodulatory or antimicrobial effects and improve technological performance in dairy matrices. Their stability, predictable functionality, and favourable safety profile make them suitable alternatives or supplements to traditional probiotics. In dairy applications, postbiotics can be produced in situ during fermentation or incorporated as formulated ingredients to enhance product functionality and deliver consistent bioactivity. In addition to highlighting the technological benefits of LAB-derived postbiotics in dairy systems and assessing new regulatory frameworks, this review summarizes the current understanding of their classification, composition, and mechanisms of action. Key challenges related to definition, standardization, safety assessment and labeling are discussed to support future development of postbiotic-enriched functional dairy foods.

## 1. Introduction

Lactic acid bacteria (LAB) are fundamental to the manufacture of fermented dairy products and have long been utilized to improve their sensory attributes, microbiological safety, and shelf life, as well as to develop dairy-based functional foods [1]. Growing consumer awareness of diet–health relationships and the global shift toward healthier lifestyles have markedly expanded the functional food market. In parallel, the consumption of dairy products enriched with health-promoting properties has increased, positioning fermented dairy matrices as major contributors to this trend [1,2].

Historically, the functional properties of fermented dairy products have been largely attributed to the presence of viable probiotic microorganisms, particularly LAB. These microorganisms may contribute to gut microbiota modulation, intestinal barrier reinforcement, and immunological regulation. However, the efficacy of probiotic-containing dairy products remains strongly influenced by strain-specific characteristics and by the ability of microorganisms to survive technological processing, storage and gastrointestinal transit. Maintaining adequate viability throughout the product shelf life is a constant challenge in industrial dairy systems and may compromise reproducibility of physiological effects in vivo. Furthermore, increasing regulatory assessment regarding probiotic health claims has intensified interest in identifying more stable and standardized bioactive alternatives [3]. Consequently, recent research has increasingly focused on postbiotics, including non-viable microbial cells, structural components, and fermentation-derived metabolites capable of exerting biological effects independently of microbial viability.

In recent years, this perspective has expanded to include a broader range of microbially derived bioactive substances produced during LAB fermentation, including metabolites and inactivated microbial components. These substances are referred to as postbiotics. Along with probiotics, prebiotics, and synbiotics, postbiotics are collectively categorized as “biotics” and are increasingly recognized for their potential to promote host health [4]. Postbiotics are described as preparations that may include inactivated microorganisms, microbial lysates, cell components, and metabolites [5]. This development reflects a conceptual shift in functional dairy science: rather than focusing exclusively on the delivery of viable microorganisms, researchers increasingly recognize that fermentation-derived metabolites, structural components, and non-viable microbial cells can exert beneficial effects and enhance product functionality, a perspective supported by emerging definitions of postbiotics that include non-viable cells and metabolic byproducts with demonstrable health effects [6].

The inclusion of postbiotics in functional food formulations including dairy products is gaining attention due to their bioactive peptide content and technological advantages such as greater stability during processing and storage, reduced safety concerns related to live microbial transfer, and consistent bioactivity independent of viability [7,8,9,10]. Moreover, research indicates that many health effects traditionally attributed to probiotics may arise not only from live cultures but also from fermentation-derived compounds and microbial components that persist after cell inactivation. This understanding has encouraged the development of dairy and other food matrices intentionally enriched with postbiotic substances produced in situ by LAB during fermentation or incorporated as formulated ingredients to confer functional benefits without reliance on microbial viability [6,7,11].

Postbiotics generally have a number of appealing qualities that are highly sought after, including clear chemical structures, safety dose criteria, and a longer shelf life when used as an ingredient in foods and beverages or as nutritional supplements [6].

Recent advances in functional dairy science highlight a clear transition from reliance on viable probiotics toward the utilization of postbiotics as stable and predictable bioactive ingredients. Postbiotics are now widely recognized as key contributors to the functional properties of fermented dairy products. Existing reviews consistently demonstrate that dairy matrices such as yoghurt, kefir, and cheese serve as effective carriers of LAB-derived postbiotics including exopolysaccharides, bioactive peptides, bacteriocins, and organic acids, which contribute to both technological improvements and biological activities. However, despite the growing body of literature, current reviews emphasize several critical gaps, including inconsistencies in definitions, limited standardization of postbiotic preparations and analytical methods [4,12,13,14,15]. Therefore, the present review aims to address these limitations by providing a comprehensive and critically structured overview of LAB-derived postbiotics in dairy systems, focusing on their classification, production, and stability, as well as their technological applications, functional properties, and regulatory challenges, in order to support the development of scientifically substantiated and industrially applicable functional dairy products.

## 2. Definitions of Postbiotics

**Figure 1:**
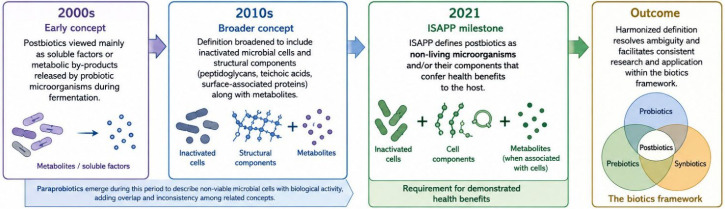
Evolution of definitions of postbiotics.

Several definitions of postbiotics have been proposed in the scientific literature, reflecting the evolution of the concept over the past two decades (Figure 1). Early references to postbiotics in the 2000s generally described them as soluble factors or metabolic by-products released by probiotic microorganisms during fermentation [16]. As research progressed throughout the 2010s, the definition broadened to include inactivated microbial cells and structural components such as peptidoglycans, teichoic acids, and surface-associated proteins, which were increasingly recognized as contributors to host health effects [6,17]. During this period, the term paraprobiotics also emerged to describe non-viable microbial cells with biological activity, adding further overlap and inconsistency among related concepts [18]. These differing interpretations are reflected in the varying definitions summarized in Table 1.

A major milestone was reached in 2021, when the International Scientific Association for Probiotics and Prebiotics (ISAPP) defined postbiotics as non-living microorganisms and/or their components that confer health benefits to the host [19], thereby harmonizing previous concepts and emphasizing the requirement for demonstrated health benefits. This definition unified earlier views by recognizing both non-viable cells and their structural constituents while requiring demonstrated health benefits as a defining criterion. As summarized in Table 1, the ISAPP formulation resolves much of the ambiguity seen in earlier interpretations and has facilitated more consistent research and application of postbiotics within the broader biotics framework, which includes probiotics, prebiotics, and synbiotics [4].

Postbiotics arise naturally during microbial fermentation, where probiotics metabolize available substrates—including prebiotic carbohydrates—into a diverse array of bioactive compounds [20,21]. The greater the availability of fermentable carbohydrates, the more varied the spectrum of postbiotic metabolites produced [20], which helps explain why earlier definitions focused on metabolites, whereas later conceptualizations incorporated the entire inactivated cell and its components.

**Table 1:** Definitions of postbiotics in the scientific literature.

Definition	Includes Inactivated Cells	Metabolites Explicitly Included in Definition	Relevance to Dairy Foods	Reference
Non-viable bacterial products or metabolic byproducts from probiotic microorganisms that have biologic activity in the host	Yes	Yes	Includes metabolites formed during dairy fermentation but lacks food-matrix specificity	Tsilingiri, Rescigno [16]
Soluble factors (products or metabolic byproducts) secreted by live bacteria or released after bacterial lysis	No (focus on soluble factors)	Yes	Excludes inactivated starter cultures; applicable to dairy whey, fermented milk, and cheese extracts	Aguilar-Toalá et al. [6]
Metabolic products of probiotic bacteria with health-promoting properties	No	Yes	Emphasizes metabolites but excludes non-viable LAB cells common in processed dairy foods	Zendeboodi et al. [18]
Functional bioactive compounds produced by food-grade microorganisms during fermentation	Not specified	Yes	Relevant to dairy fermentation but does not clearly distinguish postbiotics from general fermentation products	Collado et al. [22]
A preparation of inanimate microorganisms and/or their components that confers a health benefit on the host	Yes	Does not classify metabolites alone as postbiotics (but allows them when associated with cell structures)	Particularly relevant to fermented dairy products containing heat-treated LAB, non-viable starter cultures, and microbial cell components with demonstrated health benefits.	ISAPP [19]

Definitions reported before the 2021 ISAPP consensus frequently included microbial metabolites, soluble factors, fermentation products, or cell-free supernatants as postbiotics. However, according to the current ISAPP definition, a postbiotic must consist of a preparation of inanimate microorganisms and/or their components that confers a documented health benefit on the host. Therefore, definitions based exclusively on isolated metabolites or soluble fermentation-derived compounds do not fully conform to the current ISAPP framework. The final column indicates the relevance of each definition to dairy systems and highlights conceptual differences between historical and current terminology.

The ISAPP definition emphasizes the presence of non-viable microbial cells and/or cellular structures and distinguishes postbiotics from preparations consisting solely of purified metabolites [19]. Consequently, some researchers argue that isolated compounds such as organic acids, short-chain fatty acids (SCFAs), vitamins, exopolysaccharides, bacteriocins, and bioactive peptides should not be considered postbiotics when separated from the microbial biomass that produced them. Instead, these compounds are more accurately described as microbial metabolites or bioactive fermentation products [23].

Several related terms are frequently encountered in the literature and are often used interchangeably despite important conceptual differences (Table 2). Microbial metabolites refer to low-molecular-weight compounds generated during microbial metabolism, including organic acids, SCFAs, vitamins, peptides, and neurotransmitter-like molecules such as γ-aminobutyric acid (GABA) [24]. Fermentates generally describe complex preparations obtained from microbial fermentation that may contain viable or non-viable microorganisms, metabolites, cell fragments, and residual culture medium components [6]. Paraprobiotics are typically defined as inactivated microbial cells that confer physiological benefits without requiring viability [17], whereas metabiotics refer to structurally defined microbial-derived molecules or metabolites responsible for specific biological activities [25]. While these concepts overlap substantially, they differ in composition, degree of characterization, and proposed mechanisms of action.

The distinction between these categories remains a subject of scientific debate. Some authors advocate a broad interpretation of postbiotics that encompasses both non-viable microbial cells and their metabolic products [6,10], whereas others support the more restrictive ISAPP definition. Because much of the literature published before the ISAPP consensus used broader terminology, numerous studies continue to classify microbial metabolites and cell-free supernatants as postbiotics (Table 2). This inconsistency complicates comparisons among studies and highlights the need for greater terminological standardization [19,26].

In this review, the ISAPP consensus definition is used as the primary conceptual framework, whereby postbiotics are defined as preparations of inanimate microorganisms and/or their components that confer a health benefit on the host. Accordingly, isolated microbial metabolites such as organic acids, bacteriocins, exopolysaccharides, bioactive peptides, and γ-aminobutyric acid (GABA) are not considered postbiotics when purified and evaluated independently of microbial biomass. However, because these compounds are frequently discussed within the broader postbiotic literature and often occur as constituents of fermentation-derived preparations, they are reviewed here as postbiotic-associated bioactive compounds. This distinction is maintained throughout the manuscript to align with current scientific consensus while acknowledging historical usage of the term in food science literature [6,19].

**Table 2:** Comparison of postbiotics and related microbiome-derived concepts frequently encountered in the literature. The definitions presented reflect current scientific understanding, including the ISAPP consensus statement on postbiotics, while acknowledging the overlap that exists among these terms in older publications.

Term	Definition/Main Components	Microbial Cells Present?	Typical Composition	Example	Key References
**Postbiotics (ISAPP definition)**	Preparation of inanimate microorganisms and/or their components that confers a health benefit on the host	Yes (non-viable)	Inactivated cells, cell wall fragments, intracellular components, metabolites associated with the preparation	Heat-killed *Lacticaseibacillus rhamnosus* or *Lactiplantibacillus plantarum* preparations	Salminen et al. [19], Vinderola et al. [26]
**Microbial metabolites**	Low-molecular-weight compounds produced during microbial metabolism	No	Organic acids, SCFAs, vitamins, peptides, GABA	Lactic acid, acetate, GABA, folate	Wegh et al. [23], Żółkiewicz et al. [24]
**Fermentates**	Complex fermentation-derived preparations containing microbial products and residual fermentation components	Variable	Metabolites, cell fragments, residual medium constituents, sometimes viable or non-viable microorganisms	Fermented dairy extracts, cell-free fermentation broths	Aguilar-Toalá et al. [6]
**Paraprobiotics**	Inactivated microbial cells that provide physiological benefits without viability	Yes (non-viable)	Whole heat-killed or otherwise inactivated microbial cells	Heat-treated LAB cultures	Taverniti and Guglielmetti [17]
**Metabiotics**	Structurally defined microbial-derived molecules responsible for biological activity	No	Purified metabolites, signaling molecules, peptides, cell components	Bacteriocins, bioactive peptides, peptidoglycan fragments	Shenderov [25]

The ISAPP consensus definition excludes purified metabolites alone from the formal definition of postbiotics unless they are present as part of a preparation of inanimate microorganisms and/or their components. Consequently, compounds such as lactic acid, SCFAs, bacteriocins, vitamins, and bioactive peptides are more accurately described as microbial metabolites or metabiotics when isolated and characterized independently. Nevertheless, many studies published before and after the ISAPP consensus continue to classify such compounds as postbiotics, contributing to terminological inconsistencies across the literature.

## 3. Postbiotic Preparations and Associated Metabolites in Dairy Systems

**Figure 2:**
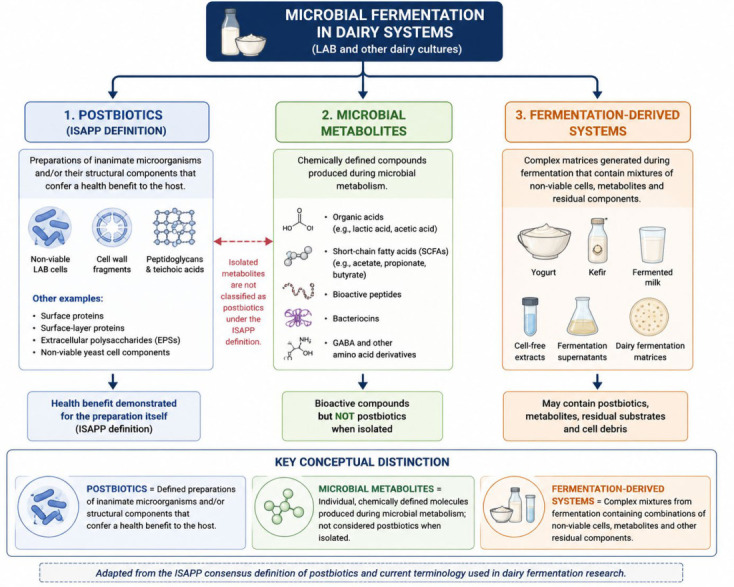
Conceptual classification of microbial-derived bioactive components in fermented dairy systems according to the ISAPP framework. Postbiotics are defined by the International Scientific Association for Probiotics and Prebiotics (ISAPP) as preparations of inanimate microorganisms and/or their components that confer a health benefit on the host and therefore include non-viable microbial cells, cell wall fragments, and cellular structures when present within a demonstrated postbiotic preparation. In contrast, microbial metabolites such as organic acids, short-chain fatty acids (SCFAs), bioactive peptides, bacteriocins, vitamins, and γ-aminobutyric acid (GABA) are not classified as postbiotics when isolated and evaluated independently, although they may be present within postbiotic preparations and contribute to their biological activity. Fermentation-derived systems (fermentates) represent broader fermentation products that may contain combinations of viable or non-viable microorganisms, metabolites, cell fragments, and residual substrate components. This classification follows the ISAPP consensus definition and is intended to distinguish postbiotics from related microbiome-derived concepts commonly grouped under broader historical definitions.

Lactic acid bacteria fermentation generates a complex spectrum of bioactive components that persist beyond microbial viability and contribute to both the technological performance and potential health effects of fermented dairy products. However, it is essential to distinguish between true postbiotic preparations, as defined by the International Scientific Association of Probiotics and Prebiotics (ISAPP), and individual metabolites or compounds that are frequently, but not consistently, referred to as postbiotics in the food science literature (Figure 2) [27].

According to the ISAPP framework, postbiotics are defined as preparations of inanimate microorganisms and/or their structural components that confer a health benefit on the host, and not as isolated metabolites alone [19]. Nevertheless, in dairy research, many studies include microbial metabolites such as organic acids, short-chain fatty acids (SCFAs), peptides and signalling molecules within a broader, pragmatic interpretation of postbiotic functionality. This dual usage introduces conceptual ambiguity and complicates comparisons across studies. Therefore, in this section, a clear distinction is maintained between postbiotic preparations (non-viable microbial cells and structural components) and postbiotic-associated metabolites (bioactive compounds produced during fermentation but not classified as postbiotics in isolation). Below, some of the main LAB-derived postbiotics and postbiotic-associated metabolites are described. Their mechanisms of action and health implications are further discussed in Section 4.

### 3.1. Organic Acids (Postbiotic-Associated Metabolites)

Although organic acids are traditionally classified as microbial metabolites, they are frequently discussed within the broader and pragmatic postbiotic framework in food science, particularly when present within complex fermentation-derived matrices. Organic acids, including lactic acid, acetic acid, and smaller amounts of propionic and formic acids, are among the most abundant and functionally significant postbiotic-associated metabolites generated during dairy fermentation. These metabolites are primarily produced by LAB, such as *Lactobacillus delbrueckii* subsp. *bulgaricus* and *Streptococcus thermophilus*, through the fermentation of lactose and other carbohydrates in milk systems. Acidification of the dairy matrix represents one of the most fundamental biochemical transformations in fermented milk products and is essential for their safety, stability, and sensory characteristics [28,29].

From a technological and microbiological perspective, organic acids play a central role in preservation by inhibiting the growth of spoilage and pathogenic microorganisms [30,31]. Within the postbiotic framework, postbiotic-associated metabolites like organic acids are particularly relevant because their functional activity persists independently of microbial viability. As stable fermentation-derived metabolites, they remain active even after heat treatment or cell inactivation, contributing to the consistent antimicrobial and technological performance of fermented dairy products [32].

Overall, organic acids represent a fundamental group of LAB-derived postbiotics-associated metabolites that contribute to microbial inhibition, product stability, and sensory characteristics in dairy systems. Their well-characterised mechanisms of action and stability make them one of the most reliable and extensively studied functional components in fermented foods [33].

### 3.2. Bioactive Peptides (Postbiotic-Associated Metabolites)

Bioactive peptides are released from milk proteins through LAB proteolytic systems during fermentation and cheese ripening. Strains such as *Lactobacillus helveticus*, *Lactococcus lactis*, and *Lacticaseibacillus plantarum* are particularly effective at generating peptides with antihypertensive [34], antioxidant and anti-inflammatory activities [35]. These peptides act through various mechanisms, including ACE inhibition, radical scavenging and immunomodulation. Their contribution to the health-promoting properties of fermented milk is recognized broadly in recent food microbiology literature, which highlights their therapeutic potential and their importance in shaping functional dairy products [15].

Peptide-based postbiotic-associated metabolites are more compatible with pasteurized or long-shelf-life dairy products, provide better safety profiles for vulnerable populations, and are more technologically stable than live probiotics. Because of these benefits, LAB-derived bioactive peptides are desirable components for the development of nutraceuticals, medical nutrition products, and functional dairy products [15].

### 3.3. Gamma-Aminobutyric Acid (GABA) (Postbiotic-Associated Metabolites)

Certain LAB possess glutamate decarboxylase (GAD) systems that enable the bioconversion of L-glutamate into γ-aminobutyric acid (GABA) during fermentation. Among these, *Lactobacillus brevis* is one of the most extensively characterized high-GABA-producing species, owing to its efficient GAD pathway and acid-resistance mechanisms. *Lactococcus lactis* has also been reported to express glutamate decarboxylase activity under specific fermentation conditions, contributing to GABA accumulation in dairy matrices [36].

During milk fermentation, the GAD system functions not only as a metabolic pathway but also as an acid stress response mechanism, consuming intracellular protons and enhancing bacterial survival under low-pH conditions. This dual physiological and technological relevance makes GABA production particularly attractive in fermented dairy systems [37].

GABA-enriched dairy products have attracted considerable attention due to their potential antihypertensive and neuroactive properties. Human intervention studies have demonstrated reductions in systolic and diastolic blood pressure following consumption of GABA-rich fermented milk products, although reported effects vary substantially depending on dosage, duration of intervention, host characteristics, and fermentation conditions [38]. In addition to cardiovascular effects, dietary GABA has been associated with stress reduction, improved sleep quality, and modulation of psychological stress markers in several clinical and experimental studies [37]. Nevertheless, important mechanistic uncertainties remain unresolved. Although GABA functions as the principal inhibitory neurotransmitter within the central nervous system, the extent to which orally consumed GABA crosses the blood–brain barrier remains controversial. Current evidence suggests that many observed physiological effects may instead involve indirect peripheral pathways, including enteric nervous system signaling, vagal nerve stimulation, gut–brain axis interactions, and modulation of neuroendocrine responses. Although the ability of orally administered GABA to directly cross the blood–brain barrier remains uncertain, several peripheral mechanisms have been proposed to explain its physiological effects. GABA receptors are expressed throughout the enteric nervous system (ENS), where GABA may influence intestinal neurotransmission, gut motility, secretion, and sensory signalling. Activation of these pathways can generate afferent signals that are transmitted to the central nervous system through the vagus nerve, thereby providing a mechanistic link between dietary GABA and brain function. In addition, GABA may influence gut–brain communication through interactions with enteroendocrine cells, stimulating the release of signalling molecules involved in neuroendocrine regulation. Emerging evidence also suggests that GABA-producing microorganisms and fermentation-derived GABA may modulate gut microbial activity and metabolite production, thereby indirectly affecting stress responses, autonomic nervous system regulation, and behavioural outcomes through gut–brain axis signalling. Together, these observations suggest that many of the reported neuroactive effects of dietary GABA are more likely mediated through peripheral gut–brain communication pathways than through substantial direct penetration of the blood–brain barrier [39,40]. Furthermore, considerable heterogeneity exists among available clinical studies regarding GABA concentration, product formulation, intervention duration, and outcome assessment, limiting direct comparison between studies.

Given its antihypertensive, neuroactive, and stress-reducing properties, GABA produced during dairy fermentation is increasingly recognized as a valuable postbiotic-associated metabolite. Unlike probiotics, its functional activity does not depend on bacterial viability, and its chemical stability allows incorporation into shelf-stable functional dairy formulations. Consequently, GABA-enriched fermented milk products represent promising next-generation functional foods targeting both neurological and cardiovascular health [41,42].

### 3.4. Bacteriocins (Postbiotic-Associated Metabolites)

Bacteriocins are ribosomally synthesised antimicrobial peptides produced during microbial metabolism. Although isolated bacteriocins do not meet the ISAPP definition of postbiotics, they are frequently present within fermentation-derived postbiotic systems and contribute to their biological activity. These proteinaceous molecules are typically secreted into the extracellular environment and exhibit inhibitory activity against foodborne pathogens and spoilage microorganisms, with either narrow or broad antimicrobial spectra depending on their structure and origin. Their primary mechanism of action involves interaction with the bacterial cell membrane, leading to pore formation, membrane permeabilisation, and leakage of intracellular contents, ultimately resulting in cell death. In some cases, bacteriocins also interfere with essential cellular processes such as cell wall biosynthesis and enzyme activity, enhancing their antimicrobial efficacy [43].

In dairy fermentation systems, bacteriocins such as nisin and pediocin are widely recognised for their effectiveness in controlling pathogenic bacteria, *including Listeria monocytogenes*, thereby improving product safety and extending shelf life. Their production during fermentation forms part of a multi-hurdle preservation system together with organic acids, hydrogen peroxide, and other antimicrobial metabolites produced by LAB. Importantly, bacteriocins fulfil key criteria for postbiotic-associated metabolites, as their biological activity is retained independently of microbial viability, allowing their application in heat-treated or processed dairy products. Due to their natural origin, specificity, and generally recognised safety, bacteriocins are increasingly considered promising alternatives to synthetic preservatives and potential tools in functional food development.

### 3.5. Exopolysaccharides (Components That May Occur Within Postbiotic Preparations)

Exopolysaccharides (EPSs) are high-molecular-weight extracellular polymers synthesized by LAB during fermentation and represent an important class of postbiotic substances in dairy systems. EPSs may be secreted into the surrounding matrix or remain associated with the bacterial cell surface as capsular polysaccharides. In fermented dairy products, EPSs are primarily produced by *Streptococcus thermophilus*, *Lactococcus lactis*, and species within the genera *Lactobacillus* and *Lacticaseibacillus*, which are widely used as starter cultures [44].

From a technological perspective, EPSs play a critical role in improving the rheological and textural properties of fermented dairy products. In yogurt and fermented milk, EPSs contribute to increased viscosity, water-holding capacity and resistance to syneresis, thereby enhancing product stability and mouthfeel. These functional properties result from interactions between EPS molecules and milk proteins, facilitating the formation of a stabilised gel network and improved structural integrity of the product. The presence of EPS-producing strains has also been shown to enhance creaminess and sensory perception in yogurt-type products. Importantly, the technological performance of EPSs depends strongly on their molecular characteristics, including molar mass, monosaccharide composition, and branching structure, which vary among strains and fermentation conditions [45,46,47].

Beyond their technological functionality, EPSs exhibit a range of biological activities, supporting their classification as components that may be included in postbiotic preparations. Increasing evidence indicates that LAB-derived EPSs can exert immunomodulatory effects through interactions with host cells. For example, EPSs from *Streptococcus thermophilus* have been shown to modulate immune responses in intestinal epithelial cells by reducing pro-inflammatory cytokine expression and regulating signalling pathways. More broadly, microbial EPSs have been reported to influence immune function, including modulation of inflammatory responses and immune signalling, partly through interactions with host receptors and regulation of cytokine production [48,49].

In addition to immunomodulation, EPSs possess multifunctional bioactive properties, including antioxidant, antimicrobial, cholesterol-lowering, and prebiotic-like effects. These prebiotic-like activities may involve stimulation of beneficial gut microbiota and modulation of microbial metabolism, contributing to improved intestinal health. EPSs are also implicated in biofilm formation and bacterial adhesion, which may enhance bacterial survival during fermentation and influence microbial interactions both in food matrices and in the gastrointestinal environment [50].

In the context of postbiotics, EPSs offer several advantages compared with live probiotic cultures. Their functional activity does not depend on microbial viability, and they exhibit high physicochemical stability during processing and storage. Furthermore, EPS-based formulations may provide more consistent functionality than live cultures, which are subject to viability loss and strain variability. However, challenges remain regarding the structural heterogeneity of EPSs, the influence of strain-specific biosynthesis pathways, and the need for improved analytical tools to establish clear structure–function relationships [51].

Overall, LAB-derived EPSs represent multifunctional postbiotic components that contribute both to the technological optimisation of fermented dairy products and to their potential health-promoting properties. Their dual role as texture-modifying agents and bioactive compounds makes them particularly attractive for the development of next-generation functional dairy foods.

### 3.6. Cell Wall Fragments (Postbiotics by ISAPP Definition)

Cell wall fragments derived from LAB are increasingly recognized as important postbiotic constituents in fermented dairy foods. During fermentation, ripening, storage or thermal processing, LAB cells may undergo autolysis or inactivation, releasing structural components such as peptidoglycans, teichoic acids, lipoteichoic acids, surface proteins, and polysaccharide fragments into the dairy matrix. These microbial cell-wall components can retain biological activity even after loss of viability and have been associated with immunomodulatory, antimicrobial, anti-inflammatory, and gut barrier-supporting effects [6,19]. Fermented dairy products such as yogurt, kefir, cheese, and cultured milk therefore represent important dietary sources of these bioactive postbiotic structures. The 2021 International Scientific Association for Probiotics and Prebiotics consensus definition of postbiotics specifically includes non-living microorganisms and their components that confer health benefits on the host, thereby encompassing cell wall fragments commonly present in heat-treated or aged fermented dairy products [19].

### 3.7. Short-Chain Fatty Acids (Postbiotic-Associated Metabolites)

Short-chain fatty acids (SCFAs), including acetate, propionate, and butyrate, are microbial metabolites that may be considered within the broader postbiotic-associated metabolite framework when present in fermentation-derived systems. In dairy matrices, SCFAs are typically produced in relatively low concentrations compared to their extensive generation in the gastrointestinal tract, with their formation depending on microbial strain composition, substrate availability, and fermentation conditions. Their production is mainly associated with heterofermentative metabolic pathways, as well as secondary processes such as citrate and amino acid metabolism. In fermented dairy products, SCFAs contribute primarily to flavour and aroma development and may support microbial stability through synergistic antimicrobial effects with organic acids. Beyond their technological relevance, SCFAs have been widely studied for their physiological functions, particularly in the context of gut health. These compounds are known to enhance intestinal barrier integrity, modulate immune responses, and exert anti-inflammatory effects, mainly through mechanisms involving G protein-coupled receptor activation and inhibition of histone deacetylases. Among them, butyrate plays a key role as an energy source for colonocytes and as a regulator of gene expression. However, it is important to note that most evidence regarding these health effects derives from SCFAs produced by the gut microbiota, and the direct contribution from dairy-derived SCFAs remains less clearly established. Overall, while SCFAs represent a biologically important class of microbial metabolites, their role in dairy systems is more closely linked to technological and sensory functions, with potential health effects largely extrapolated from gastrointestinal studies [16,24,52].

## 4. Functional Properties and Health Implications

In the past decade, LAB-derived postbiotic preparations have gained increasing recognition due to their diverse functional properties. Because these preparations frequently contain inanimate microbial cells and their structural fragments together with microbe-produced substances generated during fermentation, their biological activity is likely determined by the combined effects of various bioactive compounds [53].

Among the reported functional properties, antimicrobial activity represents an important characteristic in the food industry, supporting the potential application of LAB-derived postbiotic preparations as natural preservatives, particularly in dairy products [54,55,56,57]. Beyond contributing to the preservation of dairy foods, these preparations have also been associated with several health-related implications, including modulation of immune responses, reduction of inflammatory processes, maintenance of gut barrier integrity, and support of microbiota balance [58,59,60].

While antimicrobial effects of LAB-derived postbiotic preparations are often well documented in vitro and partially validated in food matrices, most health-related claims remain supported primarily by preclinical studies, with limited and heterogeneous clinical evidence available. This discrepancy highlights a critical gap between mechanistic insights and translational relevance.

The following sections critically evaluate the main functional properties of these preparations, with emphasis on bioactive compounds that may contribute to these effects, their proposed mechanisms of action, level of supporting evidence, and existing limitations.

### 4.1. Antimicrobial and Preservative Effects

Microbial contamination remains a major challenge in dairy production, posing a threat to the safety and quality of dairy products [61]. Dairy foods may become contaminated at various stages of the production chain, including milking, processing, packaging, transportation, and storage [62]. Therefore, effective strategies are required to control the growth of pathogenic and spoilage microorganisms in such products. Recently, LAB-derived postbiotic preparations have emerged as a promising alternative strategy for the inhibition of microbial growth in dairy foods [54,55,56,57]. Their antimicrobial activity, which contributes to the preservation of dairy products, is mainly attributed to certain metabolites retained in these preparations.

Organic acids represent a major group of metabolites responsible for the antimicrobial activity of LAB-derived postbiotic preparations in dairy products. During dairy fermentation, LAB produce a variety of organic acids, including lactic, acetic, citric, formic, malic, pyruvic, and tartaric acids [63,64]. These acids induce acidification of the extracellular environment, which promotes the diffusion of their undissociated forms across the microbial cell membrane into the cytoplasm. Within the near-neutral cytoplasm, these acids dissociate into protons and anions, leading to intracellular acidification. This process disrupts the activity of enzymes involved in cellular energy metabolism [65,66]. SCFAs, including acetate, butyrate, and propionate, are another group of metabolites exhibiting a similar antimicrobial mechanism [67,68,69]. Their undissociated forms can diffuse across microbial cell membranes and dissociate within the cytoplasm, resulting in intracellular acidification and dysregulation of cellular metabolic processes [70].

Bacteriocins also constitute a major group of metabolites involved in controlling pathogenic and spoilage microorganisms in dairy products. They typically act by binding to specific membrane-associated receptors. These receptors mainly include components of the mannose phosphotransferase system (Man-PTS). Following Man-PTS binding, bacteriocins such as lactococcin A and pediocin PA-1 cause membrane permeabilization through pore formation in the cytoplasmic membrane, leading to leakage of intracellular contents, thereby disrupting cellular homeostasis [71,72]. Furthermore, some bacteriocins, including nisin and lacticin 3147, bind to lipid II rather than membrane-associated receptors such as Man-PTS. Binding to lipid II leads not only to pore formation in the cytoplasmic membrane but also to inhibition of cell wall synthesis, as lipid II is a precursor in peptidoglycan biosynthesis [73,74].

In addition, hydrogen peroxide (H_2_O_2_), a metabolic by-product produced by LAB, contributes to the inhibition of microbial growth in dairy products. H_2_O_2_ exerts its antimicrobial activity through oxidative stress associated with the accumulation of reactive oxygen species, which damage essential cellular components, including nucleic acids, proteins, and lipids [75,76].

Current studies support the antimicrobial activity of LAB-derived postbiotic preparations, demonstrating strain-dependent effects against diverse microorganisms. These findings indicate that these preparations inhibit spoilage microorganisms and may act as natural preservatives, thereby contributing to the extension of shelf life in dairy products [55,69,77,78,79]. Moreover, they inhibit microorganisms linked to human gastrointestinal infections, highlighting their relevance for dairy safety [54,57,69,77,78,79]. Representative studies on the antimicrobial and preservative effects of LAB-derived postbiotic preparations are summarized in Table 3.

Despite the promising antimicrobial activity of LAB-derived postbiotic preparations, several limitations must be considered. Studies that fully comply with the ISAPP definition of postbiotics remain limited, as cell-free supernatants lacking inanimate microbial cells and/or cell fragments are still frequently classified as postbiotics, such studies were therefore not included in the present analysis. The available evidence suggests that the antimicrobial activity of postbiotic preparations is mainly associated with metabolites, particularly organic acids, whereas the contribution of inanimate microbial cells and cell fragments remains insufficiently understood. In addition, only a limited number of studies have investigated LAB-derived postbiotic preparations in dairy matrices, and these have been restricted to a few specific dairy products [54,55,56,57,69,77,78]. Consequently, antimicrobial efficacy observed in individual dairy products may not be directly transferable to the broader range of dairy products. Furthermore, variability in producing strains, postbiotic preparation composition, concentrations, and methodological approaches, together with the reliance on reference laboratory strains, limits the comparability and generalizability of the findings. Overall, these limitations highlight the need for standardized production and characterization protocols, as well as more complex dairy systems that better reflect industrial dairy conditions.

### 4.2. Immunomodulatory and Anti-Inflammatory Activities

Beyond their antimicrobial and preservative effects, LAB-derived postbiotic preparations obtained from dairy products possess functional properties associated with potential health benefits, particularly immunomodulatory and anti-inflammatory activities. Immunomodulatory activity refers to the ability of these preparations to regulate immune system responses through the modulation of immune cell activity, cytokine production, and immune signaling pathways. Meanwhile, anti-inflammatory effects involve the attenuation of pro-inflammatory responses, contributing to the maintenance of immune homeostasis [58,59,60,80]. These properties are largely attributed to metabolites, particularly SCFAs, and to microorganism-associated molecular patterns (MAMPs) present in LAB-derived postbiotic preparations.

SCFAs are among the most important metabolites contributing to the immunomodulatory and anti-inflammatory effects of LAB-derived postbiotic preparations. These effects are mediated through activation of G protein-coupled receptors and inhibition of histone deacetylases (HDACs), which modulate macrophage and dendritic cell functions, alter cytokine production, and promote regulatory T cell differentiation [81,82,83]. Consequently, SCFAs help maintain immune homeostasis by attenuating excessive inflammatory responses, promoting anti-inflammatory mechanisms, and supporting balanced immune function.

Peptidoglycan, lipoteichoic acids, lipoproteins, EPSs, and pili are considered prominent cell wall- and surface-associated components of LAB that act as MAMPs involved in the regulation of immune function and inflammatory processes. These MAMPs are recognized by host pattern recognition receptors (PRRs), including Toll-like receptors and NOD-like receptors, with each MAMP interacting with specific receptor types. PRRs are expressed on both immune cells (e.g., dendritic cells, lymphocytes, macrophages, and monocytes) and non-immune cells (e.g., endothelial and epithelial cells). Their activation triggers intracellular signaling cascades, predominantly involving the NF-κB and MAPK pathways, leading to modulation of immune responses through alterations in immune cell activity and cytokine production [80,84,85,86,87,88,89,90,91].

Evidence suggesting the immunomodulatory and anti-inflammatory effects of dairy-derived LAB postbiotic preparations has been reported across in vitro, animal, and human studies. In vitro studies using immune and epithelial cell lines have demonstrated that LAB-derived postbiotics modulate cytokine production, resulting in decreased production of pro-inflammatory cytokines, including TNF-α, IL-1β, IL-6, IL-8, and IL-17α, alongside increased production of anti-inflammatory cytokines such as IL-10 [3,58,80,92,93]. Notably, LAB-derived postbiotic preparations have also been shown to restore the inflammatory/anti-inflammatory balance [93]. In vivo studies using murine models have shown that LAB-derived postbiotic preparations obtained from dairy products exert immunomodulatory and anti-inflammatory effects in experimentally induced models of intestinal inflammation. Oral administration of postbiotic preparations has been associated with reduced pro-inflammatory cytokine levels and increased anti-inflammatory cytokine levels, as well as attenuation of tissue-level inflammatory responses and reduction of disease-related inflammatory markers in experimental models [59,93,94]. In addition, certain dairy-derived LAB postbiotic preparations have been reported to modulate immune cell populations, including the Th17/Treg balance, enhance NK cell activity, and increase immunoglobulin production in murine models [83]. Evidence from human studies investigating the immunomodulatory and anti-inflammatory effects of LAB-derived postbiotic preparations obtained from dairy products remains very limited, with only one study identified in the available literature. In a randomized, double-blind, placebo-controlled human trial, these preparations induced immunomodulatory effects, including a significant reduction in circulating IL-1β levels, suggesting potential anti-inflammatory activity in healthy adults [95].

The current evidence on the immunomodulatory and anti-inflammatory effects of LAB-derived postbiotic preparations obtained from dairy products remain restricted by a number of limitations, and their application in humans has not yet been fully established. Most of the available evidence is derived from in vitro and animal studies, whereas human clinical studies are extremely scarce, preventing definitive conclusions regarding their effects in humans despite their promising potential. In vitro studies often rely on simplified monoculture systems that do not fully reflect the complexity of the intestinal environment, while animal models, although informative, may not accurately replicate human immune responses [58,59,60,80,92,93,94,95]. Consequently, the translational relevance of these findings remains uncertain. Furthermore, the lack of standardization in postbiotic preparations, including differences in composition, production methods, cell models, inflammatory stimuli, and outcome measures, limits direct comparison between studies. Therefore, standardized experimental approaches and well-designed clinical studies are required to confirm the immunomodulatory and anti-inflammatory effects of LAB-derived postbiotic preparations in humans.

### 4.3. Gut Barrier Function and Microbiota Modulation

Functional properties of dairy-derived LAB postbiotic preparations with potential implications for human health include not only immunomodulatory and anti-inflammatory activities, but also the maintenance of gut barrier function and modulation of the gut microbiota. These preparations may contribute to gut barrier integrity by enhancing the expression of tight junction proteins, stimulating mucus production, supporting epithelial cell function, and attenuating intestinal inflammation [59,60,93,96]. Moreover, they modulate the gut microbiota by suppressing pathogenic and promoting beneficial microorganisms, thereby contributing to a more balanced microbial community [59,97]. These functional properties are primarily linked to microbial metabolites, among which SCFAs are considered the most significant, as well as MAMPs.

SCFAs, as LAB-derived metabolites, enhance intestinal barrier integrity by increasing the expression of tight junction proteins, including occludin, claudins, and zonula occludens (ZO) proteins, which connect neighboring epithelial cells, seal intercellular spaces, and regulate the selective permeability of the intestinal epithelium. This effect is primarily mediated through epigenetic mechanisms involving the inhibition of HDACs, thereby promoting the transcription of genes associated with the formation of tight junctions [98,99]. Additionally, SCFAs reduce inflammatory signaling through inhibition of NF-κB activation, thus limiting pro-inflammatory cytokine-mediated disruption of epithelial junctions [100]. Beyond their contribution to gut barrier function, they also participate in microbiota modulation via luminal acidification, which suppresses acid-sensitive pathogenic microorganisms and promotes the growth of beneficial commensal bacteria [101].

In addition to SCFAs, other LAB-derived metabolites, including bacteriocins and organic acids, contribute to gut barrier and microbiota-related effects by inhibiting pathogenic microorganisms, thereby promoting a balanced intestinal microbial community and indirectly supporting intestinal barrier integrity through reduced risk of inflammation-associated epithelial damage [102,103,104].

Gut barrier function and microbiota modulation are also influenced by MAMPs derived from LAB. Among these, EPSs are especially relevant, as they enhance intestinal barrier integrity by strengthening tight junction proteins, promoting mucus production, and modulating immune responses, thereby attenuating epithelial permeability [105]. In addition, EPSs may be fermented by the gut microbiota, facilitating cross-feeding interactions that promote the growth of beneficial bacteria [97]. Other MAMPs, including peptidoglycan and lipoteichoic acids, may indirectly contribute to gut barrier modulation through interactions with PRRs, leading to modulation of immune signaling, which may support epithelial tight junction stability and mucosal immune homeostasis [84,85,86].

Evidence from in vitro and animal studies, together with limited findings from human studies, indicates the potential functional roles of LAB-derived postbiotic preparations obtained from dairy products in gut health. In vitro studies using intestinal epithelial models, including Caco-2 and IEC-6 cells, have demonstrated that these preparations improve intestinal barrier function, as reflected by increased transepithelial electrical resistance and enhanced expression of tight junction proteins such as claudin-1, occludin, and ZO-1 [60,93,96,106]. Animal studies, particularly in murine intestinal inflammation models, further support these findings, demonstrating that dairy-derived LAB postbiotic preparations improve gut barrier function. Improved gut barrier function has been associated with reduced intestinal permeability, enhanced expression of tight junction proteins, restoration of goblet cell populations, enhanced mucin production, and attenuation of intestinal mucosal damage. These effects are commonly accompanied by modulation of gut microbiota composition, including recovery of microbial homeostasis [59,87,93,97,105]. Evidence from human studies remains scarce, although emerging findings have been reported. Available studies suggest that LAB-derived postbiotic preparations obtained from dairy products may modulate gut microbiota composition and support gastrointestinal function. These changes have been associated with shifts toward a more balanced microbial profile alongside improvements in gastrointestinal outcomes [95,107].

The limitations observed in studies on gut barrier function and microbiota modulation are largely similar to those reported for the immunomodulatory and anti-inflammatory effects of dairy-derived LAB postbiotic preparations, with the most prominent limitation related to the very limited number of human clinical studies. Evidence is predominantly derived from in vitro and animal models, while human data are restricted to only a few microbiota-focused studies, highlighting a significant gap in clinically validated evidence. Therefore, further well-designed human studies are needed to confirm these effects in humans.

## 5. Production and Stability of Postbiotics in Dairy Matrices

The production and stability of LAB-derived postbiotics in dairy matrices depend on several interrelated factors, including fermentation conditions, starter culture selection, and processing parameters. Conditions during fermentation influence the production of bioactive compounds, including both cellular components and microbial metabolites, in postbiotic preparations, thereby modulating their functional activity [108,109,110,111]. In parallel, starter culture selection contributes to the determination of the specific functional properties of LAB-derived postbiotic preparations due to strain-specific abundance of cellular structures, together with metabolic capacities, leading to the formation of distinct bioactive compounds. Processing parameters may affect cell inactivation, the stability of these compounds, and the overall functionality of postbiotic preparations [112,113,114]. Collectively, these factors affect the composition, stability, and functional properties of LAB-derived postbiotic preparations in dairy matrices. These aspects are discussed in detail in the following sections.

### 5.1. Influence of Fermentation Conditions and Starter Culture Selection

Fermentation by LAB is a dynamic metabolic process that leads to the formation of diverse bioactive compounds in dairy matrices through the metabolism of nutrients in the substrate. These compounds persist beyond microbial viability and constitute the basis of the functionality of LAB-derived postbiotic preparations [11,21]. The production of bioactive compounds is strongly influenced by fermentation parameters and the selection of starter cultures, which determine their composition and concentration in the postbiotic preparations.

Among fermentation parameters, temperature is one of the most critical factors, as it directly regulates LAB metabolic activity. When temperature deviates from the optimal range, metabolic pathways are altered, thereby affecting both the rate and profile of bioactive compounds production. Suboptimal temperatures may slow growth while simultaneously enhancing the accumulation of compounds associated with stress adaptation, including bacteriocins and EPSs. Importantly, the optimal growth temperature of LAB does not necessarily coincide with the highest formation of bioactive compounds, which is often promoted under suboptimal or mildly stressful conditions [108,109,110]. For example, *Pediococcus acidilactici* CCFM18 showed peak bacteriocin production at 32 °C, despite higher temperatures being more favorable for growth, suggesting that mild thermal stress may enhance bacteriocin production [110]. However, temperature effects are highly strain-dependent, as some LAB strains do not exhibit significant differences in bacteriocin formation between optimal and suboptimal growth temperatures [108].

Alongside temperature, pH represents another important parameter influencing the formation of bioactive compounds by LAB. As observed for temperature, suboptimal pH conditions may induce acid stress responses that modulate metabolic activity and can promote the formation of stress-associated compounds such as bacteriocins and EPSs, whereas excessive acid stress may suppress their synthesis by disrupting cellular metabolism [108,115].

Oxygen availability may further influence the production of bioactive compounds through its effects on cellular metabolism and adaptive responses to oxidative stress. Although LAB primarily rely on fermentative metabolism and generally do not require oxygen for growth, oxygen exposure may modify metabolic pathways and affect the production of these compounds [116]. Controlled oxygen conditions have been reported to affect the formation of bacteriocins and EPSs, with responses varying depending on strain characteristics and cultivation conditions [108,111]. Consequently, oxygen availability may contribute to variations in the yield and composition of bioactive compounds.

Fermentation duration has been reported to play an important role in determining the yield and composition of bioactive compounds produced by LAB. The accumulation of these is closely associated with microbial growth phase and metabolic state, with production typically occurring during the exponential phase and reaching maximum levels toward the late exponential or stationary phase [108,117]. Extended fermentation may enhance the release of bioactive compounds. However, excessively long fermentation can also lead to the degradation of previously formed compounds and a reduction in bioactivity due to excessive acidification [118].

The production of bioactive compounds is influenced not only by fermentation conditions but also by the choice of starter cultures, as different strains exhibit distinct metabolic characteristics that determine fermentation results. These differences in metabolic activity directly affect both the amount and compositional diversity of bioactive compounds formed during fermentation. For instance, *Lactococcus lactis* is widely recognised for its ability to produce nisin, a well-characterised bacteriocin that is commercially used as a natural food preservative and contributes to microbial safety in fermented dairy systems [112,113]. In contrast, *Streptococcus thermophilus* is notable for its EPS production, which has been associated with potential immunomodulatory and anti-inflammatory activities, as well as beneficial effects on gut barrier function and modulation of the intestinal microbiota [114]. It should also be noted that different starter cultures may exhibit structural variations that can contribute to their functional properties. *Lacticaseibacillus rhamnosus* GG, in turn, is characterised by the presence of pili, which may act as MAMPs and are suggested to contribute to host–microbe interactions involved in immunomodulatory and anti-inflammatory responses [91].

### 5.2. Processing Factors

Following fermentation, processing is closely associated with the inactivation of viable cells and the production of postbiotic preparations. Among processing factors, heat treatment is particularly important, as it is widely used to inactivate microbial cells and can therefore serve as a key technological step in postbiotic production. At the same time, thermal exposure may affect the retention and stability of bioactive compounds through structural modifications, thereby influencing their functional activity [119].

In dairy manufacturing, several thermal processing methods are applied, including low-temperature long-time pasteurization (65 °C for 30 min), high-temperature short-time pasteurization (72 °C for 15 s), extended shelf-life pasteurization (120–130 °C for 1–4 s), ultra-high-temperature sterilization (136–145 °C for  2–8 s), and in-container sterilization (112 °C for 15 min) [120]. Although these heat treatments are primarily employed to ensure microbiological safety and extend the shelf life of dairy products, they may also be applied as microbial inactivation strategies to produce LAB-derived postbiotics in situ. Thermal inactivation is therefore widely applied in postbiotic production due to its simplicity, scalability, and compatibility with existing dairy technologies, with treatment conditions ranging from mild pasteurization to more intensive sterilization regimes. These processes induce irreversible damage to essential cellular structures, resulting in the loss of microbial viability while allowing partial retention of bioactive compounds formed during fermentation [19]. Consequently, the effectiveness of thermal processing depends not only on achieving microbial inactivation but also on maintaining the balance between cell inactivation and the preservation of bioactive compounds responsible for postbiotic functionality [19,119].

The stability of bioactive compounds during thermal processing varies considerably depending on their intrinsic properties. Organic acids and SCFAs are generally highly stable under typical dairy processing conditions due to their resistance to commonly applied heat treatments [121,122]. EPSs also exhibit relatively high stability under conventional pasteurization and may therefore retain biological activity following thermal processing. Nevertheless, more intensive treatments, such as ultra-high-temperature treatment or sterilization, may induce structural alterations that may reduce their functional properties and bioactivity [123,124]. Cell wall-associated components, including peptidoglycans and teichoic acids, are typically more resistant to conventional pasteurization conditions and may therefore retain bioactivity following processing. By contrast, proteinaceous compounds such as bacteriocins exhibit variable thermal stability, which depends strongly on amino acid composition, sequence, and structural properties [125,126]. For example, nisin is considered relatively heat-stable, especially in acidic environments, whereas other bacteriocins may be more susceptible to thermal inactivation, resulting in partial loss of bioactivity under more severe thermal conditions [125,127].

Storage conditions further influence the stability and functional integrity of LAB-derived postbiotics. During storage, they may undergo physicochemical and biochemical changes that affect their functional performance. Temperature is one of the most important storage parameters determining postbiotic stability. Refrigerated conditions generally slow down degradative reactions, including proteolysis, lipid oxidation, and Maillard-related modifications, thereby preserving the activity of bioactive compounds [121,128,129,130]. In contrast, elevated storage temperatures can accelerate the degradation of proteinaceous compounds, including bacteriocins, as well as induce structural modifications in EPSs [131]. Increased temperatures may also promote interactions with milk proteins and lipids, potentially altering the stability and functional availability of postbiotics [132]. Although frozen storage is widely used for the long-term preservation of dairy products, ice crystal formation and freeze-concentration effects may induce structural alterations in dairy matrices, potentially affecting postbiotics [133,134].

### 5.3. Stability and Bioavailability in Dairy Products

The stability and bioavailability of LAB-derived postbiotics in dairy products are determined by matrix composition, processing conditions, and storage-related interactions. Stability refers to the ability of postbiotics to maintain their structural integrity and retain their bioactivity during processing and storage [135]. In dairy products, postbiotics are present within a complex matrix composed of proteins, lipids, carbohydrates, and minerals [136]. The complex composition of dairy matrices may enhance the stability of certain postbiotics by reducing their exposure to conditions leading to degradation during processing and storage. In particular, casein micelles and whey proteins may interact with bioactive compounds, thereby improving their oxidative stability and resistance to oxidative and hydrolytic degradation [137,138].

Although bioavailability describes the accessibility and biological activity of LAB-derived postbiotics from the dairy matrix within the gastrointestinal tract, it is not solely dependent on their stability but also on their release behavior during gastrointestinal digestion [135]. The dairy matrix may influence the release of bioactive compounds through the modulation of protein and lipid digestion under gastric and intestinal conditions [139,140]. During gastrointestinal digestion, postbiotics may undergo structural modifications induced by enzymatic activity and pH variations [141]. While some bioactive compounds, such as organic acids, may remain stable throughout gastrointestinal transit, others may undergo partial degradation into smaller fragments that may still retain biological activity [6]. Overall, these processes determine the extent to which postbiotics become bioaccessible and retain their biological activity at the intestinal level, thereby influencing their potential functional effects in the host.

Despite increasing interest in LAB-derived postbiotics, systematic and mechanistic studies addressing their stability, bioavailability, and storage behaviour within complex dairy matrices remain limited. Current evidence is still fragmented, as it often focuses on isolated compounds or specific processing conditions, which restricts a comprehensive understanding of how matrix interactions, processing parameters, storage conditions, and gastrointestinal environments collectively influence their functional behaviour. This highlights the need for more integrated studies linking structural stability and biological activity across processing, storage, and digestion stages.

## 6. Safety Considerations

LAB-derived postbiotics are gaining popularity as safe bioactive preparations with potential applications in food preservation, nutraceuticals, and therapeutics. Postbiotics, unlike probiotics, do not contain viable microorganisms, which reduces the risk of microbial translocation, infection, or instability during storage. Nonetheless, comprehensive toxicological assessment is still required to ensure their safety for human consumption and industrial applications [19,53,142,143].

### 6.1. Toxicological Aspects of LAB-Derived Postbiotics

The toxicological profile of LAB-derived postbiotics varies according to the producing strain, fermentation conditions, metabolite composition, purification processes, dosage, and route of administration. Organic acids, bacteriocins, peptides, exopolysaccharides, and cell wall fragments are all considered safe at recommended concentrations [6,19].

Excessive accumulation of certain metabolites can have negative biological effects [144,145]. Most studies on the cytotoxic and genotoxic potential of LAB-derived postbiotics found low cytotoxicity and no mutagenic or genotoxic effects, indicating their biocompatibility and safety. Furthermore, animal studies have demonstrated minimal adverse effects on body weight, organ histology, hematological indices, and liver or kidney function after postbiotic administration [146].

Despite these positive findings, some LAB strains may produce undesirable metabolites such as biogenic amines, including histamine and tyramine, which can cause allergic reactions, headaches, and hypertension when consumed in large quantities. Furthermore, high concentrations of acidic metabolites or bacteriocins can disrupt intestinal microbiota balance or cause mucosal irritation. Therefore, careful strain selection and controlled fermentation processes are critical to ensuring product safety [145]. To reduce the risk of biogenic amine formation, candidate LAB strains should be screened for amino acid decarboxylase genes (e.g., hdc, tdc, and odc) using PCR or whole-genome sequencing. Molecular screening should be complemented by phenotypic assessment of biogenic amine production, ensuring that only non-decarboxylase-producing strains are selected for safe postbiotic production in dairy fermentation [119].

Another significant aspect is the immunomodulatory activity of postbiotics. While many LAB-derived metabolites have anti-inflammatory properties, overstimulation of immune responses in susceptible people cannot be completely avoided. As a result, more long-term and clinical safety studies are needed, especially for vulnerable populations like infants, the elderly, and immunocompromised patients [19,53].

Overall, current evidence indicates that LAB-derived postbiotics have a favorable safety profile and a low toxicological risk [147]. However, standardized safety evaluation protocols and regulatory guidelines are still required to facilitate their widespread use in the food and biomedical industries.

### 6.2. Antibiotic Resistance Gene Concerns Associated with LAB-Derived Postbiotics

Although LAB-derived postbiotics are generally regarded as safe, there have been concerns raised about the presence and transfer of antibiotic resistance genes (ARGs) associated with the producer strains. Many LAB strains used in food fermentation and postbiotic production naturally harbor intrinsic or acquired resistance determinants, which can contribute to the spread of antimicrobial resistance if not properly evaluated [148,149].

Even though postbiotics do not contain viable microorganisms, residual cellular components, extracellular vesicles, or free DNA fragments carrying ARGs may remain in the final product, especially if insufficient purification or inactivation procedures are used. Horizontal gene transfer mechanisms, such as transformation, transduction, and conjugation, pose theoretical risks for the transmission of resistance genes to commensal or pathogenic microorganisms within the gastrointestinal tract [150,151].

The occurrence of transferable resistance genes in certain LAB strains, including resistance to tetracycline, erythromycin, chloramphenicol, and vancomycin, has been described, and these genes are often associated with mobile genetic elements such as plasmids and transposons, increasing the likelihood of dissemination under favorable conditions [152]. Therefore, strain-specific genomic screening and safety assessment are essential prior to the industrial application of LAB-derived postbiotics.

Regulatory agencies, such as the European Food Safety Authority and the Food and Drug Administration, emphasize the importance of assessing antimicrobial resistance profiles in microbial products intended for food or therapeutic use. Whole-genome sequencing and molecular characterization are being recommended for identifying acquired and transferable ARGs as well as ensuring safety compliance [19,153].

Overall, while the risk associated with antibiotic resistance transfer from LAB-derived postbiotics is thought to be lower than that from live probiotic preparations, stringent quality control, purification procedures, and genomic surveillance are still required to reduce potential safety concerns.

### 6.3. Dosage and Long-Term Consumption Concerns of LAB-Derived Postbiotics

The safety and efficacy of LAB-derived postbiotics are highly dependent on the dosage and duration of use. Although postbiotics are generally considered safe, excessive intake or prolonged exposure may potentially induce unwanted physiological or metabolic effects [6,19].

Appropriate dosage levels are especially important because postbiotics contain a wide range of bioactive compounds, such as organic acids, bacteriocins, peptides, and microbial cell components, the biological activities of which can vary depending on concentration. Acidic metabolite concentrations may cause gastrointestinal discomfort, mucosal irritation, or disruptions in intestinal homeostasis. Similarly, excessive exposure to bacteriocins or other antimicrobial metabolites may change the composition of beneficial gut microbiota and affect microbial balance [144].

Long-term consumption of postbiotics is also an area that requires further investigation. While short-term studies generally show favorable safety profiles, there is little information available about chronic exposure and cumulative biological effects. Continuous intake may influence immune responses, metabolic pathways, or host–microbiome interactions over time, particularly in vulnerable populations such as infants, the elderly, pregnant women, and immunocompromised patients [53,154].

Another concern associated with LAB-derived postbiotics is the possible accumulation of fermentation-derived metabolites, including biogenic amines and other secondary compounds, particularly when unsuitable strains or inadequate manufacturing conditions are employed. Since these metabolites originate from microbial fermentation processes, their presence in postbiotic preparations may contribute to adverse effects such as allergic reactions, headaches, hypertension, or metabolic disturbances in susceptible individuals [155,156].

## 7. Regulatory and Commercial Challenges

Despite increasing scientific and commercial interest in postbiotics, significant regulatory and standardization challenges continue to limit their widespread incorporation into functional dairy products. One of the primary obstacles involves the absence of globally harmonized definitions and regulatory frameworks governing postbiotic-containing foods. Although the ISAPP consensus definition has provided conceptual clarification, regulatory authorities in different jurisdictions continue to apply varying interpretations regarding classification, safety assessment, and permitted health claims [19].

Within the European Union, the European Food Safety Authority (EFSA) applies strict requirements for substantiating health claims associated with functional food ingredients. Demonstration of causality between postbiotic consumption and specific physiological benefits requires robust human clinical evidence, which remains limited for many proposed postbiotic preparations [19]. Furthermore, variability in postbiotic composition arising from differences in microbial strains, fermentation substrates, processing conditions, and inactivation methods complicates standardization and reproducibility [10]. In addition, the lack of harmonized quality control standards, including specifications for batch-to-batch consistency, minimum effective dosage, and analytical characterization of bioactive components, remains a major barrier to regulatory approval and commercialization [26,157].

In the United States, regulatory evaluation may additionally involve Generally Recognized as Safe (GRAS) status assessments, particularly for novel postbiotic preparations or concentrated microbial fractions intended for commercial food applications [19]. In contrast, Japan regulates postbiotic-containing products under broader functional food frameworks, whereas specific postbiotic regulations are still evolving in countries such as China, Brazil and Argentina, highlighting differences in global regulatory approaches [158]. Challenges also arise regarding labeling practices because consumers may not clearly distinguish postbiotics from probiotics or fermented ingredients [23].

Another important challenge concerns manufacturing consistency and analytical characterization. Unlike purified pharmaceutical compounds, postbiotic preparations frequently contain complex mixtures of metabolites, peptides, polysaccharides, and microbial structural components whose composition may vary significantly between production batches. Establishing validated analytical methods, dose standardization criteria, and stability parameters therefore remains essential for future regulatory approval and commercial scalability [10,19].

Future regulatory progress will likely depend on improved standardization strategies, well-designed clinical trials, and internationally harmonized definitions capable of distinguishing postbiotics from related concepts such as paraprobiotics, metabiotics, and fermented food ingredients [158,159,160].

## 8. Conclusions

The rapid expansion of postbiotic research reflects a broader transition in microbiome science from an exclusive focus on microbial viability toward a greater understanding of fermentation-derived biological functionality. Within dairy systems, LAB-derived postbiotic preparations offer a particularly promising approach for combining technological advantages with potential health-promoting effects while avoiding several challenges associated with maintaining probiotic viability throughout processing, storage and gastrointestinal transit. Despite this, the field remains at a critical stage of development. The lack of universally accepted definitions, variability in production processes, insufficient standardization of compositional characterization, limited human clinical evidence and unresolved regulatory questions continue to restrict scientific comparability and commercial implementation. Overcoming these barriers will require a shift from descriptive research toward mechanism-driven and evidence-based investigations. Future studies should place greater emphasis on identifying the specific bioactive components responsible for health effects, establishing clear dose–response relationships and validating efficacy through well-designed and adequately powered clinical trials. Stronger interdisciplinary collaboration among microbiologists, food technologists, clinicians, regulatory authorities and industry stakeholders will be essential for translating promising laboratory findings into commercially viable products. Harmonized analytical methodologies and internationally recognized regulatory frameworks should be prioritized to ensure consistency, reproducibility and consumer confidence. Furthermore, integrating postbiotic development into existing dairy manufacturing systems may offer a practical and economically feasible pathway for accelerating market adoption.

Overall, postbiotics should not be viewed solely as an alternative to probiotics but as a distinct and scientifically evolving category of functional ingredients with unique technological and biological attributes. If current scientific and regulatory challenges are addressed through coordinated international efforts, postbiotic-enriched dairy foods will become an important component of the next generation of evidence-based functional foods, contributing to both product innovation and public health nutrition.

## Figures and Tables

**Table 3:** Antimicrobial and preservative activities of LAB-derived postbiotic preparations against pathogenic and spoilage microorganisms relevant to dairy products.

LAB Strain	Postbiotic Production Method	Postbiotic Preparation	Main Bioactive Compounds Linked to Antimicrobial Activity	Target Microorganisms	Reported Effect	Level of Evidence	Reference
*Lactiplantibacillus plantarum* 299v	Thermal inactivation or thermo-ultrasonic treatment, followed by cooling	Thermally and thermo-ultrasonically inactivated whole-cell culture postbiotic preparation	Organic acids	*Escherichia coli*, *Listeria monocytogenes*, *Salmonella enterica* serovar Typhimurium, *Salmonella enterica* serovar Enteritidis, *Shigella sonnei*, *Staphylococcus aureus*	Variable in vitro antimicrobial activity against Gram-positive and Gram-negative pathogens, with the strongest inhibition zones observed for thermo-ultrasonically treated postbiotics against *Listeria monocytogenes*, *Salmonella* Typhimurium, *Shigella sonnei*, and *Staphylococcus aureus*, while complete loss of antimicrobial activity after neutralization indicated acid-mediated inhibitory effects	In vitro	Vera-Santander et al. [79]
Multiple LAB strains belonging to the genera *Lactobacillus*, *Lactococcus*, *Lactiplantibacillus*, *Levilactobacillus*, *Loigolactobacillus*, *Enterococcus*, and *Streptococcus*	Centrifugation to separate supernatant and cells pellets, sonication of cell pellets, re-centrifugation, membrane filtration	Mixture of cell-free extract and cell wall/surface components	Organic acids	*Bacillus cereus*, *Escherichia coli*, *Mycobacterium tuberculosis*, *Salmonella enterocolitica*, *Staphylococcus aureus*	Variable in vitro antimicrobial activity against Gram-negative and Gram-positive bacteria, depending on the LAB strain, along with strain-dependent reductions in *Escherichia coli* counts during storage of pasteurized milk, indicating highly variable antimicrobial performance determined by strain	In vitro, dairy matrix	Tariq et al. [15]
*Ligilactobacillus salivarius* LSA-6	Thermal inactivation followed by freeze-drying	Thermally inactivated whole-cell culture postbiotic preparation	Organic acids	*Listeria monocytogenes*	Strong antibacterial activity against *Listeria monocytogenes* with dose-dependent bactericidal effects, induced membrane disruption, cytoplasmic leakage, and oxidative stress, highly effective under in vitro conditions, but showed reduced efficacy in cheese due to food matrix interactions	In vitro, dairy matrix	Shi et al. [56]
Multiple LAB strains belonging to the genera *Lacticaseibacillus*, *Latilactobacillus*, *Lactiplantibacillus*, *Leuconostoc*, *Levilactobacillus*, and *Weissella*	Thermal inactivation, pasteurization, high-pressure treatment, or sonication, followed by centrifugation to separate supernatant and cell pellets	Cell-free supernatant and treated bacterial cell pellet fractions	Carbonyl compounds, α-dicarbonyl compounds, alcohols, and sulfur-containing volatiles	*Listeria monocytogenes*, *Staphylococcus aureus*	Variable, strain-dependent antimicrobial activity observed in vitro and in cheese, with activity restricted to *Staphylococcus aureus* and strongest inhibition achieved by pascalization-derived postbiotics; while sonication-, pasteurization-, and sterilization-derived preparations showed no inhibitory effects, pellet fractions alone exhibited no antimicrobial activity, indicating that the observed bioactivity was associated with metabolites present in the cell-free supernatant rather than cellular debris	In vitro, dairy matrix	Gajewska et al. [57]

## Data Availability

No new data were created or analyzed in this study. Data sharing is not applicable to this article.
